# Cyclopædia of Practice of Medicine

**Published:** 1875-04

**Authors:** 


*Cyclopædia of the Practice of Medicine.* Edited by Dr. H. von Ziemssen. Vol. II. Acute Infectious Diseases. American Translation Edited by Albert H. Buck, M. D., New York. New York: Wm. Wood & Co., 1875.

The promise given in the first volume of this work is fully sustained by the value of the second, which is a continuation of the subject considered in the first. The topics considered by Prof. Thomas are Varicella, Measles, Rubeola, and Scarlet Fever. These are ably treated ; of Scarlet Fever he says that isolation should be strictly practiced, and goes so far as to suggest that the authorities should see that this is enforced. Beyond isolation of the sick he does not consider any other prophylactic measures of any value ; in this he takes pains to include a denunciation of Belladonna. He calls the symptomatic treatment the only rational one, and impresses upon his reader the importance of closely watching every feature of the disease. He announces his conviction of the value of bathing, and recommends the baths of Ziemssen during the high fever incident to the early stages of the attack.

Small Pox receives an able consideration from the pen of Dr. Curschmann. The history and various stages of the disease are well described. The treatment advised is one which will meet with the approval of all. As prophylactic measures complete isolation, as far as possible, of the infected, vaccination and re-vaccination are the only means recommended, of course the destruction or complete disinfection of all articles used around the patient should be insured.

In Erysipelas Dr. Zuelzer recommends the employment of the expectant treatment, a stimulating course being pursued in cachectic patients, or those suffering from other serious diseases. In serious forms, with high fever, the mineral acids in conjunction with from three to five grains of quinine several times a day are employed. Local treatment he does not seem to attach much importance to; cold baths, however, he says, may be sometimes employed with benefit. Miliary Fever, Dengue, Influenza and Hay Fever constitute the remaining portions written by Dr. Zuelzer.

Malarial Diseases receive full consideration from Prof. Hertz, and the volume closes with an article upon Cerebro-Spinal Meningitis by Prof. von Ziemssen. This the author considers that he is sustained by facts in ranking with infectious diseases. Under the head of Pathological Anatomy he says that the lesions observable are quite constant. The most important changes are found almost without exception at every autopsy. In the only *post-mortem* out of some fifty cases which we had the opportunity of seeing in the course of a few months, no pathological changes could be found, and in none of the cases was there any evidence that would lead to an idea of their infectious origin. The general description of the disease is good. The measures recommended for treatment are not new, but will be seen to be all that can be advised in our present knowledge of the etiology of Cerebro-Spinal Meningitis.

As a whole, the volume will form a valuable portion of the work when completed. Vol. III., which will be published shortly, will be upon Chronic Infectious Diseases.

